# On African Eupsilobiinae (Coleoptera: Endomychidae) with Descriptions of a New Genus and Species

**DOI:** 10.1673/031.011.16601

**Published:** 2011-12-06

**Authors:** Wioletta Tomaszewska

**Affiliations:** Museum and Institute of Zoology, Polish Academy of Sciences, Wilcza 64, 00-679 Warszawa, Poland

**Keywords:** beetles, Cucujoidea, South Africa, taxonomy

## Abstract

Species of the South African genus *Microxenus* Wollaston are revised. *Microxenus laticollis* Wollaston is redescribed, and *M. muelleri* sp. nov. and *M. krugeri* sp. nov. are described. *Natalinus* gen. nov. and its single included species, *N. klimaszewskii* sp. nov. are described. All of these taxa are diagnosed and illustrated, and a key to the species of *Microxenus* is presented. Female genitalia of newly described species are discussed in terms of monophyly of Eupsilobiinae. Zoogeographical and biological data of African Eupsilobiinae are summarized.

## Introduction

Casey established the tribe Eupsilobiini in 1895 for his new species *Eupsilobius politus.* Historically it has been difficult to place among the Cerylonid Series families. Sen Gupta and Crowson (1973) classified Eupsilobiini in Cerylonidae, and synonymized *Eupsilobius* with *Eidoreus,* established in Erotylidae by Sharp (1885) for *E. minutus* Sharp from Hawaii. Crowson (1981) suggested the relationship of *Eidoreus* with Coccinellidae, but Sasaji (1986, 1987) placed it in Endomychidae and established Eidoreinae as a new subfamily, being unaware that the name Eupsilobiini of Casey was available. Pakaluk and Ślipiński (1990) followed Sasaji in the family placement and reviewed the subfamily Eupsilobiinae at the genus and species level.

Phylogenetic studies of the family Endomychidae based on adult and adult and larval morphology combined (Tomaszewska 2000, 2005) confirmed Eupsilobiinae as members of Endomychidae. Based on these studies, Eupsilobiinae forms a distinct, monophyletic group based on adult synapomorphies like median lobe coiled apically and ovipositor with stiff, inflated, infundibulum-like structure between the bursa copulatrix and the sperm duct. However, they are also characterized by having antennal grooves on the head and a median lobe with a T-shaped basal capsule (similar to that in Coccinellidae). The single known larva of Eupsilobiinae (*Evolocera* Sharp 1902) has a rigid tooth-like prostheca like in *Mycetaea* Stephens 1829 (Mycetaeinae). These phylogenetic analyses have not demonstrated any clear relationships between Eupsilobiinae and any other subfamily of Endomychidae.

Shockley et al. (2009b) listed 6 genera and 13 species of the subfamily. Five genera are restricted to small endemic areas of Central and South America (4 genera) and South Africa (1 genus), while *Eidoreus* is known from widely scattered islands like Cuba, Guadeloupe, Virgin Islands, Galapagos, Mascarene Islands, Seychelles, Sri Lanka, Fiji, French Polynesia, Solomon Islands, and Hawaii; *E. politus* (Casey 1895) was also collected in the Florida Keys.

So far, only the genus *Microxenus* was known from the Afrotropical region, established by Wollaston (1861) for *M. laticollis* from South Africa and placed in the family Mycetophagidae. Csiki (1905, 1910) placed this genus in the endomychid subfamily Mycetaeinae, and Strohecker (1953) followed this arrangement in his world catalogue of Endomychidae. Subsequently, Pakaluk and Ślipiński (1990) placed *Microxenus* in the subfamily Eupsilobiinae, and this placement was later confirmed by phylogenetic analyses (Tomaszewska 2000, 2005).

While studying additional material of Endomychidae from South Africa, two new species of the genus *Microxenus* and a new genus of Eupsilobiinae were discovered. These taxa are described here as follows: *M. mueller*i sp. nov., *M. krugeri* sp. nov., and *Natalinus* gen. nov. with its single new species *N. klimaszewskii* sp. nov. This work raises the number of known eupsilobiine species from the Afrotropical region to 4 and the known genera to 2.

## Materials and Methods

Acronyms for depositories of specimens are:

NHM — The Natural History Museum, London, England
MIZ — Museum and Institute of Zoology PAS, Warszawa, Poland
TMNH — Transvaal Museum of Natural History, Pretoria, South Africa

Measurements were made using an ocular micrometer attached to an Olympus SZH-10 (www.olympus.com) dissecting microscope. Measurements recorded were as follows: total length from apical margin of clypeus to apex of elytra; pronotal length from the middle of anterior margin to base of pronotum; pronotal width at the widest part; elytral length along suture, including scutellum; and elytral width across both elytra at the widest part. Male and female genitalia were dissected, cleared in 10% solution of KOH, and placed in glycerine on slides for further study. Illustrations were made from slide preparations using a camera lucida attached to the same Olympus dissecting microscope.

Scanning electron micrographs photographs were made using a Hitachi S-3400N (www.hitachi.com), and digital photographs were made using a Leica digital camera (us.leica-camera. com) mounted on microscope and subsequently enhanced using Auto Montage software in the Electron Microscopy Laboratory of the MIZ.

Terminology used in this paper follows Tomaszewska (2010).

## Results

### Genus and species descriptions

**Figures 1–4.**
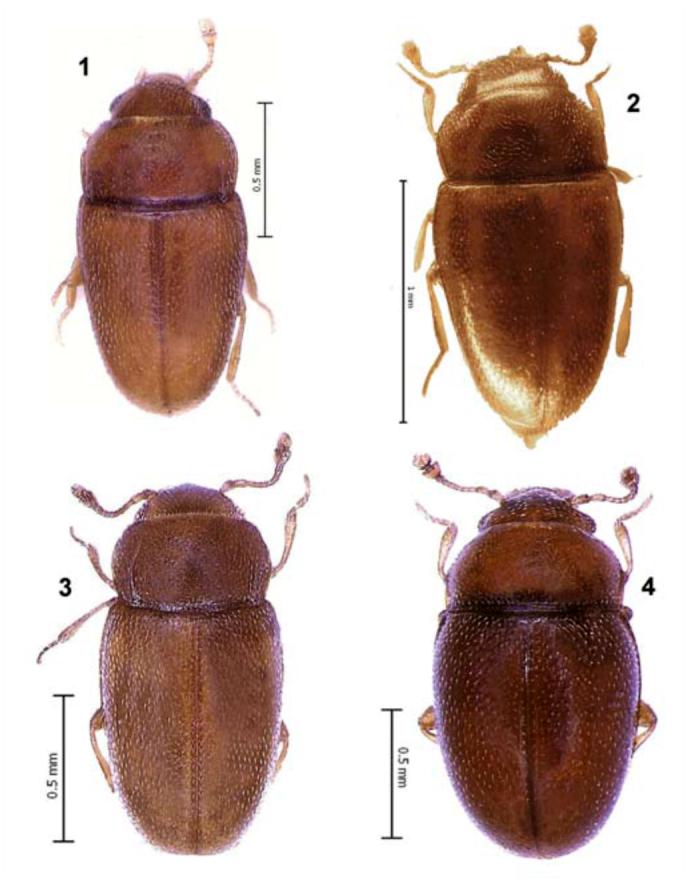
Habitus, dorsal. I: *Microxenus laticollis*; 2: *Microxenus krugeri* sp. nov.; 3: *Microxenus muelleri* sp. nov.; 4: *Natalinus klimaszewskii* gen. nov., sp. nov. High quality figures are available online.

**Figures 5–13.**
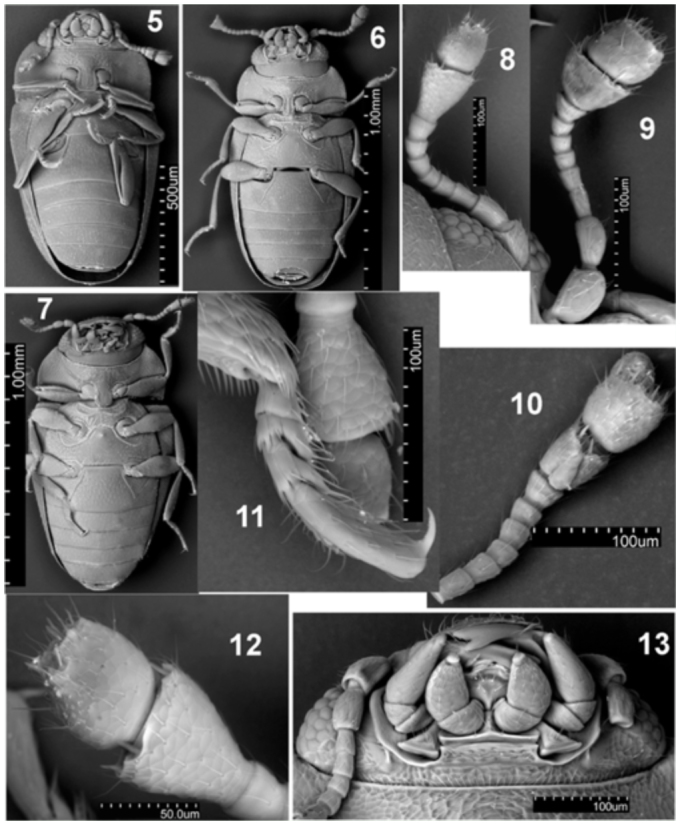
5, 10, 13: *Microxenus laticollis;* 6, 8, 11, 12: *Microxenus muelleri* sp. nov.; 7, 9: *Microxenus krugeri* sp. nov.; 5–7: habitus, ventral view; 8–10: antenna; 11: tarsus; 12: antennal club; 13: head, ventral view. High quality figures are available online.

**Figures 14–19.**
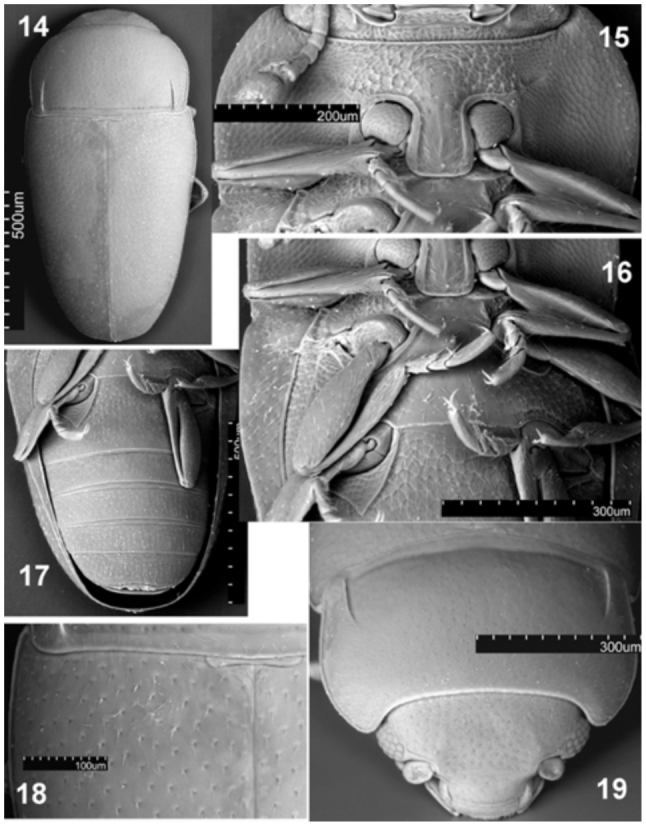
*Microxenus laticollis.* 14: habitus, dorsal; 15: prothorax, ventral view; 16: meso- and metathorax, ventral view; 17: male abdomen, ventral view; 18: prothorax and elytron connection and scutellum; 19: head and prothorax, antero-dorsal view. High quality figures are available online.

**Figures 20–25.**
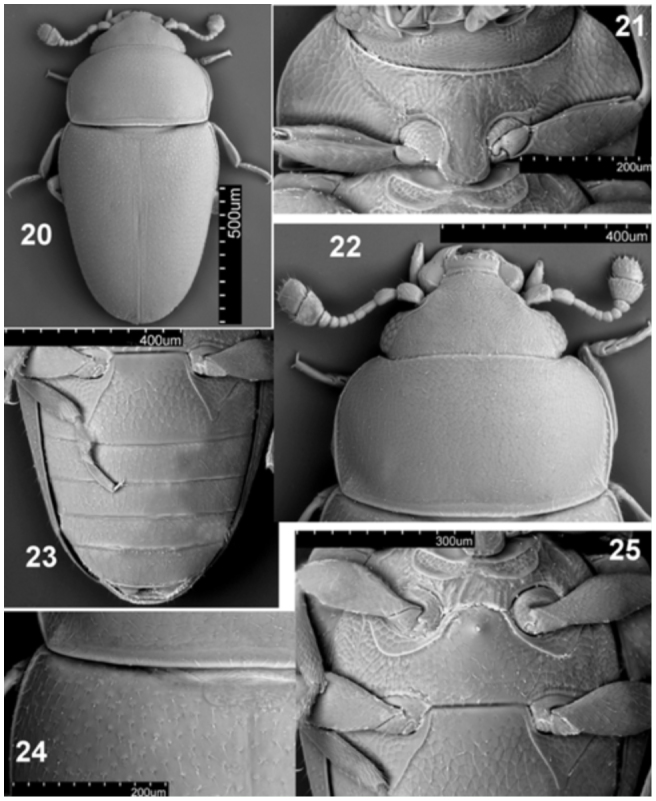
*Microxenus krugeri* sp. nov. 20: habitus, dorsal; 21: prothorax, ventral view; 22: head and prothorax, dorsal view; 23: male abdomen, ventral view; 24: prothorax and elytron connection and scutellum; 25: meso-, metathorax, and first abdominal ventrite. High quality figures are available online.

**Figures 26–32.**
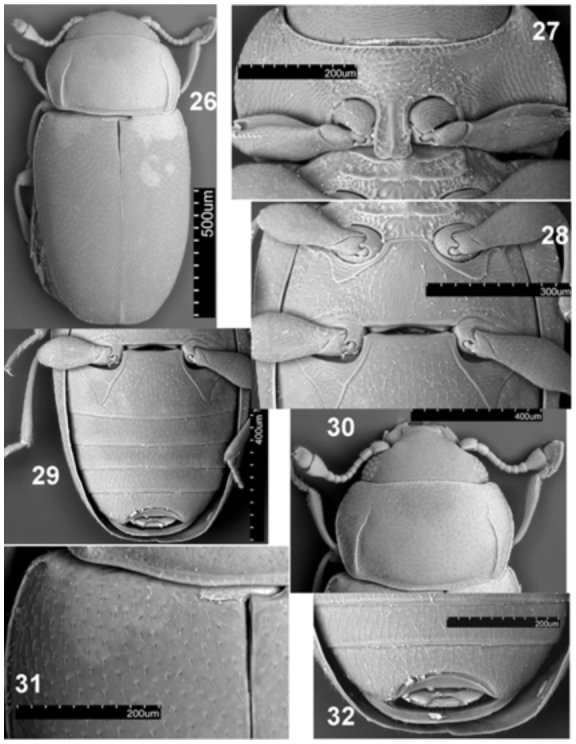
*Microxenus muelleri* sp. nov. 26: habitus, dorsal; 27: prothorax, ventral view; 28: meso-, metathorax and first abdominal ventrite; 29: maie abdomen, ventral view; 30: head and prothorax, dorsal view; 31: prothorax and elytron connection and scutellum; 32: apex of male abdomen, ventral. High quality figures are available online.

**Figures 33–41.**
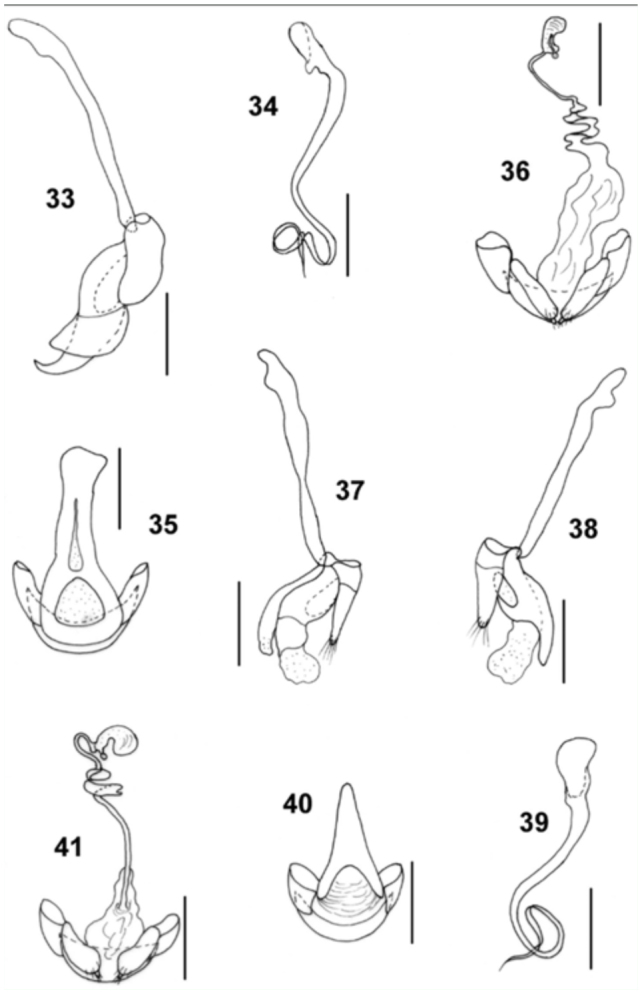
33-36: *Microxenus muelleri* sp. nov.; 37–41: *Microxenus krugeri* sp. nov.; 33, 37: tegmen, ventral view; 38: tegmen, dorsal view; 34, 39: median lobe; 35, 40: male genital segment, ventral view; 36, 41: female genitalia. Scale bar = 0.1 mm. High quality figures are available online.

**Genus *Microxenus* Wollaston (1861)**
*Microxenus* Wollaston 1861: 139. Type species, by monotypy: *Microxenus laticollis* Wollaston 1861. Pakaluk and Ślipinski 1990: 720–721 (redescription); Tomaszewska (2000) pp. 463–464 (redescription).
**Diagnosis**
  *Microxenus* is closely related to *Natalinus*. It differs from *Natalinus* by having the metaventrite with postcoxal lines, the mesoand metaventrite without postcoxal pits and the scutellum distinctly much more transverse, with at least weakly emarginate hind margin. The scutellum, which is at least 3 times wider than it is long, with weakly emarginate hind margin, is unique for *Microxenus* within Eupsilobiinae.
**Description**
  Length 1.20–1.45 mm. Body (Figures 1–3, 5–7, 14, 20, 26) is long-oval, gradually narrowing from about half of the body length to elytral apex; moderately convex; brown, shiny, smooth, covered with sparse and short pubescence (Figures 1–3).
  Head transverse and rather coarsely punctate. Gular sutures short, convergent anteriorly, widely separated. Eyes moderately large, weakly oval, prominent, coarsely-faceted. Antennal groove weakly impressed, short, expanding to posterior edge of eye (Figure 13); antennal sockets visible from above (Figures 19, 22, 30). Antenna (Figures 8–10, 12) reaches to about half the length of prothorax, 10-segmented with two-segmented club; club segments with elongate, membranous sensilla (Figures 9, 10, 12). Fronto-clypeal suture weakly arcuate. Mandible with arcuately curved outer edge; bifid at apex (Figure 13), with one small tooth on incisor edge; mola moderately large, transversely ridged; prostheca fringed; submola very small, membranous. Maxilla (Figure 13) with palpomeres 1 and 3 very short; terminal palpomere longer than remaining palpomeres combined, tapering apically. Galea blunt, with long, apical setae; twice as wide as lacinia. Lacinia with a few apical spines and setae on inner edge. Labium (Figure 13) with terminal palpomere stout and oval. Mentum trapezoidal with raised area medially. Tentorium with anterior arms fused medially and widely divergent anteriorly; corpotentorium curved (see Tomaszewska 2000: 501).
  Pronotum (Figures 19, 22, 30) transverse; pronotal surface finely and sparsely punctate; lateral margin narrowly bordered; basal sulcus distinct; lateral sulci absent or present. Prosternai process (Figures 15, 21, 27) wide with apex truncate or slightly rounded; extends posteriorly beyond procoxae. Scutellum (Figures 18, 24, 31) very small, strongly transverse, with weakly emarginate posterior margin. Mesoventrite (Figures 16, 25, 28) smooth, flat; intercoxal process at least about as wide as mesocoxal diameter. Elytron elongate, tapering, convex, with lateral margin partly visible from above; punctures irregular; epipleuron incomplete at apex (Figures 17, 23, 29). Metaventrite (Figures 16, 25, 28) strongly transverse; discrimen absent; femoral lines complete; postcoxal pits absent. Hind wing absent.
  Legs with oblique trochanterofemoral attachment; femur swollen; tibia gradually widening distally, apex surrounded by stout spines; tarsal formula 4-4-4 in both sexes 1. (Figure 11), tarsomere 2 and 3 weakly lobed ventrally, tarsomere 4 as long as remaining tarsomeres combined. Claws simple. Empodium small, bisetose.
  Abdomen (Figures 17, 23, 29) with five freely -. articulated ventrites; ventrite I with v-shaped and complete postcoxal lines; male ventrite VI subtruncate or emarginate.
  Aedeagus with median lobe sclerotized, very long, thin, coiled apically, with T-shaped capsule at base (Figures 34, 39); tegmen with basal piece at least weakly asymmetrical (Figures 33, 37, 38), with tegminal strut very long and articulated; parameres fused.
  Female genitalia with ovipositor weakly sclerotized; coxites elongate, styli small, terminal; spermatheca very small, membranous, elongate with round apex; accessory gland extremely small, elongate, membranous; sperm duct rather long, slender; basal or median part of sperm duct stiff, characteristically broad and flat (Figures 36, 41).
  **Note.** The redescription of *Microxenus* in Pakaluk and ślipiński (1990), based on specimens from South Africa and Mexico, is a mixture of features of two genera. After many years of extensive study on Endomychidae, including 2 large phylogenetic papers illustrating many different taxa (Tomaszewska 2000, 2005), it is obvious that the Mexican specimens from Pakaluk and Ślipiński (1990) do not belong to *Microxenus,* but instead most likely belong to *Evolocera,* a genus transferred from Merophysiinae to Eupsilobiinae by Tomaszewska (2005).

**Key to the species of *Microxenus***
1. Pronotum with lateral sulci long, extending at least along ⅔ of pronotal length (Figure 30); intercoxal process of mesoventrite as wide as mesocoxal diameter (Figure 28); abdominal ventrite V in male deeply emarginate (Figure 29) *M. muelleri* sp. nov.
-. Pronotum with lateral sulci absent or short, extending at most along ½ of pronotal length (Figures 14, 22); intercoxal process of mesoventrite distinctly wider than mesocoxal diameter (Figures 16, 25); abdominal ventrite V in male at most weakly emarginate (Figures 17,23) 2
2. Lateral pronotal sulci absent (Figure 22); prosternai process about as wide as procoxal diameter (Figure 21); abdominal postcoxal lines deeper, reaching more than half length of ventrite I (Figure 23); abdominal ventrite V in male subtruncate at apex (Figure 23) *M. krugeri* sp. nov.
-. Lateral pronotal sulci present (Figure 14); prosternai process wider than procoxal diameter (Figure 15); abdominal postcoxal lines shallower, reaching less than half length of ventrite I (Figure 17); abdominal ventrite V in male weakly emarginate (Figure 17) *M. laticollis* Wollaston

### Species treatments

***Microxenus laticollis* Wollaston 1861**
*Microxenus laticollis* Wollaston 1861: 140.
**Material examined**
  **Lectotype.** South Africa “*Microxenus laticollis* Woll. (type), C. of Good Hope/ Lectotype, *M. laticollis* Woll. J. Pakaluk and A. Slipinski, 1989/Syntype/Type/Lectotype” (NHM). Lectotype designation by Pakaluk and Slipinski (1990).
**Other material.** South Africa, H.E. Turner, 1920-318, Lion's Head, Cape Town, 11– 13.VII.1920 (4: NHM; 1, dissected on slide: MIZ).
**Diagnosis**
  This species is most similar to *M. krugeri* by body shape, but can be separated by the following combination of characters: short lateral sulci present on pronotum, prosternai process distinctly wider than procoxal diameter, abdominal postcoxal lines shallow and extending posteriorly less than half the length of ventrite I, and male abdominal ventrite V emarginate.
**Redescription**
  Length 1.2–1.3 mm. Body (Figures 1, 5, 14) 1.9–2.0 times as long as wide; pronotum 0.55– 0.58 times as long as wide; elytra 1.24–1.27 times as long as wide; 2.28–2.32 times longer than pronotum, 1.07–1.08 times wider than pronotum. Color reddish brown; shiny; vestiture pale, slightly denser ventrally than on dorsum. Antenna (Figure 10) with terminal segment longer than penultimate segment. Pronotum (Figures 14, 19) widest near mid-length; base slightly narrower than base of elytra; hind angles nearly right-angled; lateral sulci short, extending ⅓ of pronotal basal length; prosternai process bordered laterally; weakly expanded posteriorly, truncate at apex; about 1.30–1.35 times wider than procoxal diameter. Mesoventral process (Figure 16) 1.30–1.35 times as wide as mesocoxal diameter; elytra with lateral margins only visible from above along basal ⅓ of length (Figure 14); abdominal postcoxal lines shallow, reaching less than half length of ventrite I (Figure 17); male ventrite V weakly emarginate (Figure 17). Male and female genitalia same as in Tomaszewska (2000).
**Distribution**
  South Africa.

***Microxenus krugeri* sp. nov.**
**Material examined**
**Type Material.** Holotype (male): “S. Afr.: Kruger Nat. Pk., Pumbe sands, 24.13 S 31.56 E/ 24.1.1995, E-Y: 3096, groundtraps, leg. Endrody-Younga/ groundtraps with meat bait/ Holotype *M. krugeri* Tomaszewska” (TMNH). Paratypes: same data as holotype (9: TMNH; 4 plus 2 dissected on slide: MIZ), “S. Afr.: Kruger Nat. Pk., Skukuza, 5 km ENE, 24.59 S, 31.39 E/ 23.1.1995, E-Y: 3092, groundtraps, leg. Endrody-Younga/groundtraps with banana bait” (1: TMNH).
**Etymology**
  The specific epithet refers to Kruger National Park in the Republic of South Africa, the type locality of this new species.
**Diagnosis**
  *Microxenus krugeri* resembles *M. laticollis* in body shape, but can be separated by the following combination of characters: lateral sulci absent from pronotum, prosternai process as wide as procoxal diameter, abdominal postcoxal lines deep and extending posteriorly more than half the length of ventrite I, and male abdominal ventrite V subtruncate.
**Description**
  Length 1.20–1.25 mm. Body (Figures 2, 7, 20) 1.93–2.02 times as long as wide; pronotum 0.55–0.57 times as long as wide; elytra 1.20– 1.28 times as long as wide; 2.35–2.50 times longer than pronotum, 1.10–1.13 times wider than pronotum. Color brown with appendages lighter; vestiture pale, about as dense ventrally as on dorsum. Antenna (Figures 9, 22) with terminal segment about as long as penultimate one. Pronotum (Figure 22) widest near ⅓ of basal length; base almost as wide as base of elytra; hind angles blunt; lateral sulci absent; prosternai process (Figure 21) bordered laterally; parallel-sided, weakly rounded at apex; scarcely wider than procoxal diameter. Mesoventral process (Figure 25) 1.15–1.20 times as wide as mesocoxal diameter; elytra with lateral margins only visible above along basal ⅓ of their length (Figure 20); abdominal postcoxal lines reaching beyond half length of ventrite I (Figure 23); male ventrite V weakly rounded (Figure 23). Male genital segment (IX) and aedeagus as in Figures 37–40. Female genitalia as in Figure 41.
**Distribution**
  South Africa.

***Microxenus muelleri* sp. nov.**
**Material examined**
  **Type Material.** Holotype (male): “S. Afr., SW Cape, Kline Klipheuwe, 32.14 S, 18.26 E/ 26.8.1981, E-Y: 1851, groundtraps, 63 days, leg. Endrody-Younga/ groundtraps with feces bait/ Holotype *Microxenus muelleri* Tomaszewska” (TMNH). Paratypes: same data as holotype (8: TMNH; 2: MIZ); same but groundtraps with meat bait (1: TMNH); “S. Afr., SW Cape, Elands Bay forestry, 32.18 S, 18.21 E/ 28.8.1981; E-Y: 1853, groundtraps, 60 days, leg. Endrody-Younga/ groundtraps with banana bait” (4: TMNH), same but groundtraps with meat bait (8: TMNH; 2 plus 2 dissected on slide: MIZ); “S. Afr., SW Cape, Grootdrif farm, 32.24 S, 18.27 E/ 29.8.1981, E-Y: 1862, groundtraps, 61 days, leg. Endrody-Younga/groundtraps with meat bait” (4: TMNH; 1: MIZ), same but groundtraps with feces bait (4: TMNH; 1: MIZ); “S. Afr., SW Cape, Lamberts Bay, E, 32.05 S, 18.19 E/ 25.8.1981; E-Y: 1849, groudtraps, leg. Endrody-Younga/groundtraps with meat bait” (6: TMNH; 2: MIZ); same but groundtraps with farm. Banana bait (2: TMNH); same but 32.04 S, 18.24 E (1: TMNH); “S. Afr., SW Cape, Seweputs farm, 31.39 S, 18.22 E/ 23.8 1981, E-Y: 1835, groundtraps, leg. Endrody-Younga/groundtraps with feces bait” (2: TMNH); “S. Afr., SW Cape, Kliphoutkop, 32.17 S, 18.24 E/ 26.8.1981, E-Y: 1852, groundtraps, 60 days, leg. Endrody-Younga” (1: TMNH); same and groundtraps with banana bait” (1: TMNH); same but groundtraps with meat bait” (1: TMNH); same but groundtraps with feces bait” (1: TMNH; 1: MIZ); “S. Afr., Cape, Cedertg. Jeep track, 900 m, 32.28 S, 19.15 E/ 1.9.1981, E-Y: 1882, groundtraps, 63 days, leg. Endrody-Younga/ groundtraps with meat bait” (1: TMNH); “S. Afr., SW Cape, Cape Columbine, 32.49 S, 17.51 E/22.8 1983, E-Y: 1964, groundtraps, 73 days, leg. Endrody, Penrith/groundtraps with meat bait” (LTMNH).
**Etymology**
  The name of this new species is dedicated to Dr. Ruth Müller, a curator of the Coleoptera collection, Transvaal Museum, Pretoria (Republic of South Africa).
**Diagnosis**
  This is a very distinctive species of *Microxenus* by its regularly long-oval body, very long lateral sulci on the pronotum extending at least ⅔ length of pronotum, prosternai process with longitudinal, median carina, and male abdominal ventrite V deeply emarginate with additional submarginal groove running parallel to the emargination.
**Description**
  Length 1.20–1.45 mm. Body (Figures 3, 6, 26) 2.0–2.2 times as long as wide; pronotum 0.58– 0.63 times as long as wide; elytra 1.32–1.38 times as long as wide; 2.52–2.60 times longer than pronotum, 1.13–1.19 times wider than pronotum. Color brown with appendages slightly lighter; vestiture pale, about as dense ventrally as on dorsum. Antenna (Figures 8, 12, 30) with terminal segment shorter than penultimate one. Pronotum (Figure 30) widest near ⅓ of apical length, with base distinctly narrower than base of elytra; hind angles blunt; lateral sulci on pronotum very long, extending ⅔ of pronotal length; prosternai process (Figure 27) weakly bordered laterally, with weak, longitudinal median carina; nearly parallel-sided, truncate at apex; 0.8–0.9 times as wide as procoxal diameter. Mesoventral process (Figure 28) about as wide as mesocoxal diameter; elytra with lateral margins visible from above along nearly half of their basal length (Figure 26); abdominal postcoxal lines reaching well beyond half length of ventrite I (Figure 29); male ventrite V deeply emarginate with additional submarginal groove parallel to this emargination (Figure 32). Male genital segment and aedeagus same as in Figures 33– 35. Female genitalia same as in Figure 36.
**Distribution**
  South Africa.

**Figures 42–50.**
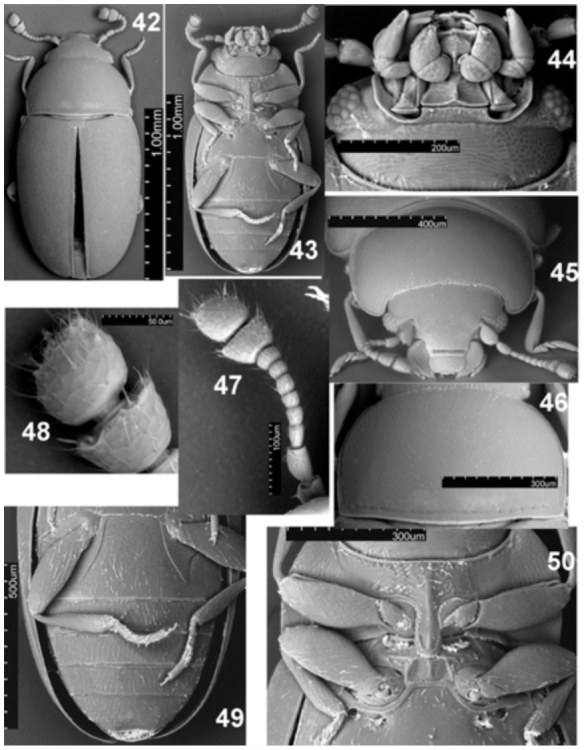
*Natalinus klimaszewskii* gen. nov., sp. nov. 42: habitus, dorsal; 43: habitus, ventral; 44: head, ventral view; 45: head and prothorax, antero-dorsal view; 46: prothorax, dorsal view; 47: antenna; 48: antennal club; 49: male abdomen, ventral view; 50: pro, meso- and metathorax, ventral. High quality figures are available online.

**Figures 51–54.**
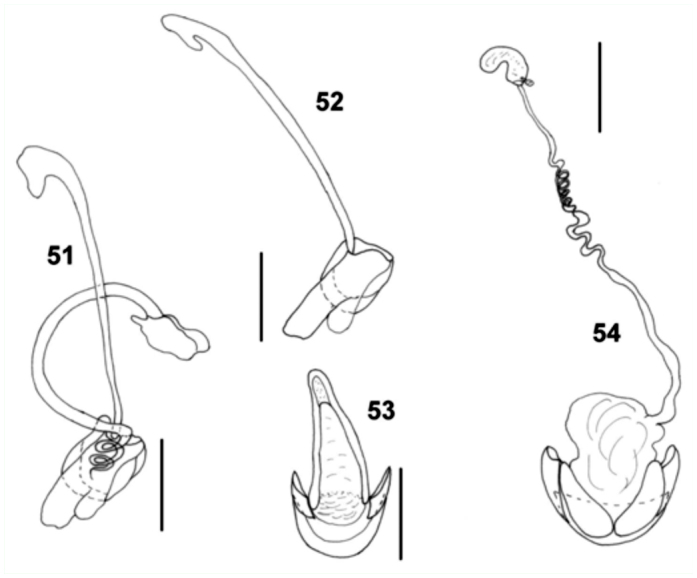
*Natalinus klimaszewskii* gen. nov., sp. nov. 51: aedeagus, ventral view; 52: tegmen, ventral view; 53: male genital segment, ventral view; 54: female genitalia. Scale bar = 0.1 mm. High quality figures are available online.

***Natalinus* gen. nov. (Figures 4, 42–54)**
**Etymology**
  The genus name refers to the Natal province in Republic of South Africa, the type locality of the type species. Gender masculine.
**Type species**
  *Natalinus klimaszewskii* sp. n.
**Diagnosis**
  *Natalinus* is apparently closely related to *Microxenus.* It can be separated by having the metaventrite without postcoxal lines, the meso- and metaventrite with large, setose postcoxal pits and the scutellum distinctly less transverse with regularly rounded hind margin. The lack of postcoxal lines within Eupsilobiinae occurs only in *Ibicarella* from Brazil, but it has 11-segmented antennae (10-segmented in *Natalinus*) and hypomeron with long, deep antennal grooves (absent in *Natalinus*). Postcoxal pits on meso- and metaventrite is unique for *Natalinus*.
**Description**
  Length 1.40–1.57 mm. Body (Figures 4, 42, 43) regularly long-oval; moderately convex; reddish brown, strongly shiny, smooth, covered with sparse and short pubescence.
  Head (Figures 44, 45) transverse, sparsely punctate. Gular sutures short, convergent anteriorly, widely separated. Eyes comparatively small, weakly oval, distinctly prominent, coarsely faceted. Antennal groove short, weakly impressed, with apex expanding to posterior edge of eye; antennal sockets visible from above. Antenna reaching ⅓ of basal length of prothorax, 10-segmented with two-segmented club (Figures 47, 48), club segments bearing elongate, membranous sensilla. Fronto-clypeal suture arcuate. Clypeus weakly transverse, flat, very weakly convergent anteriorly, truncate at apex. Labrum covered with short setae and with longer setae at sides of apex; apex membranous medially, truncate to weakly emarginate; tormae like in *Microxenus*, with mesal arms recurved anteriorly (see Tomaszewska 2000: 506). Mandible with strongly, arcuately curved lateral margin; shallowly bifid at apex (Figure 44) with one very small, blunt subapical tooth; mola large, transversely ridged; prostheca fringed; submola very small, membranous. Maxilla (Figure 44) with palpomeres 1 and 3 very short; palpomere 2 about twice as long as 1 or 3; terminal palpomere longer than remaining palpomeres combined, tapering, rounded at apex. Galea blunt, moderately wide with long, apical setae; twice as wide as lacinia. Lacinia long, narrow, with few apical and meso-apical spines. Labium (Figure 44) with palpomere 1 smallest; terminal palpomere longer than 1 and 2 combined, stout, oval, weakly truncate at apex. Mentum trapezoidal, with raised triangular area posteromedially. Prementum nearly as long as wide, sclerotized with ligula membranous, bearing weak lateral lobes, covered with long tufts of setae. Tentorium like that in *Microxenus,* with anterior arms fused medially and widely divergent anteriorly, and corpotentorium curved.
  Pronotum (Figures 42, 46) transverse, widest from base to about mid-length with base about as wide as base of elytra; pronotal surface finely and sparsely punctate; lateral margins visible throughout; basal sulcus rather distinct, lateral sulci absent; anterior angles rounded; posterior angles nearly right-angled. Pronotal disc convex. Prosternai process (Figure 50) moderately wide, bordered along lateral margins, with weak longitudinal, median carina extending anterior of procoxae nearly to anterior margin; very weakly expanded apically, with apex rounded; extending posteriorly well beyond procoxae. Procoxae circular in outline, procoxal cavity externally open, internally closed; trochantin concealed. Scutellum (Figure 42) very small, transverse, rounded posteriorly. Mesoventrite (Figure 50) with a pair of large, setose pits anterolaterally, just posterior of procoxal cavities; intercoxal process bordered, somewhat trapezoidal in shape with weak longitudinal, median carina, slightly narrower than mesocoxal diameter, not extending beyond them posteriorly. Mesocoxae circular in outline, mesocoxal cavity outwardly open; trochantin exposed. Meso-metaventral junction of straight-line type. Elytron (Figure 42) regularly elongate-oval, with rounded apex, abruptly convex, with lateral margin visible from above along half of basal length; punctation irregular; suturai stria visible from mid-length to apex; epipleuron incomplete apically (Figures 43, 49). Metaventrite (Figures 43, 50) strongly transverse, weakly convex, with two pairs of large, setose postcoxal pits; medial pit directly posterior to midpoint of mesocoxal cavity, lateral pit directly posterior to the lateral margin of the mesocoxal cavity; femoral lines absent; discrimen absent. Metacoxae transverse, widely separated. Metendosternite like that in *Microxenus*, with very short stalk and widely separated anterior arms and tendons (see Tomaszewska 2000: 526). Hind wing absent.
  Legs with oblique trochanterofemoral attachment; femur swollen; tibia gradually widening distally, apex surrounded by stout spines; tarsal formula 4-4-4 in both sexes, tarsomere 2 and 3 weakly lobed ventrally, tarsomere 4 as long as remaining tarsomeres combined. Claws simple; empodium small, bisetose.
  Abdomen (Figure 49) with five freely articulated ventrites; ventrite I nearly as long as the following three together, with long v-shaped complete femoral lines; ventrites II–IV equal in length; male ventrite V emarginate. Male abdominal segment IX lightly sclerotized (Figure 53).
  Aedeagus (Figure 51) with median lobe sclerotized, long, thin, coiled apically, with T-shaped capsule at base. Tegmen (Figure 52) short, with tegminal strut very long and articulated; parameres fused, asymmetrical apically.
  Female genitalia (Figure 54) with weakly sclerotized ovipositor, separated, elongate coxites; styli absent; spermatheca small, membranous, somewhat bean-shaped; accessory gland extremely small, elongateoval, membranous; sperm duct very long, wider in basal ⅔*,* narrower near spermatheca and weakly sclerotized, spirally twisted between ¼ of apical length to mid-length.

***Natalinus klimaszewskii* sp. nov.**
**Material examined**
  **Type Material.** Holotype (male): “S. Afr.; S. Natal, Weza, lower Stinkwood for., 30.34 S, 29.43 E / 17.11.1989; E-Y; 2686, sifted forest litter, Endrody and Klimaszewski/ Holotype *Natalinus klimaszewskii* Tomaszewska” (TMNH). Paratypes: same data as holotype (7: TMNH; 2 plus 2 completely dissected on slide: MIZ).
**Etymology**
  The name of this new species is dedicated to Jan Klimaszewski, a Polish entomologist and one of the collectors of the type series.
**Description**
  Length 1.40–1.57 mm. Body (Figures 4, 42, 43) 2.00–2.06 times as long as wide; pronotum 0.61–0.63 times as long as wide; elytra 1.26– 1.31 times as long as wide; 2.35–2.58 times longer than pronotum, 1.15–1.20 times wider than pronotum. Color reddish brown with appendages slightly lighter. Antenna (Figures 47, 48) ∼ 0.25 times as long as body; scape and pedicel large and stout; antennomere 3 slightly elongate; antennomeres 4–7 at most as long as wide; antennomere 8 strongly transverse; terminal antennomere slightly longer than penultimate one. Vestiture pale, slightly denser ventrally than on dorsum. Prosternai process (Figure 50) between coxae 0.60–0.65 times as wide as procoxal diameter; mesoventral process ∼ 0.85 times as wide as mesocoxal diameter (Figure 50); abdominal postcoxal lines reaching well beyond half length of ventrite I posterior to metacoxae (Figure 49); male ventrite V weakly emarginate (Figure 49). Aedeagus as in Figures 51, 52. Female genitalia as in Figure 54.
**Distribution**
  South Africa

## Discussion

### Systematics

Eupsilobiinae is a well-supported clade in cladistic analyses based on adult characters (Tomaszewska 2000) and on adult and larval characters combined (Tomaszewska 2005). The following two adult synapomorphies have supported monophyly of this subfamily: median lobe long, thin with T-shaped capsule at base and coiled apically, and female genitalia with infundibulum-like structure (stiff, inflated structure between bursa copulatrix and a proper sperm duct).

The present study confirmed both of these characters, although with slight modification regarding the structure of the female genitalia. The term “infundibulum-like” structure used previously by Tomaszewska (2000, 2005) is outdated and inaccurate. The term suggests that this modified structure is located exactly between the bursa copulatrix and the sperm duct, or is formed from an apical part of the bursa fused with the outlet of the sperm duct. However, female genitalia of *M. muelleri* suggest that this structure may be entirely a modification of the proper sperm duct; the genitalia of *N. klimaszewski* show less inflation in this structure and indicate that it may be formed from a sclerotized part of the sperm duct forming a twisted, rigid spiral.

The African Eupsilobiinae now includes two endemic genera: *Microxenus* and *Natalinus* gen. nov. The presence of large, setose pits on meso- and metaventrite is so far unique for *Natalinus,* while the scutellum at least three times wider than it is long, with weakly emarginate hind margin is unique for *Microxenus* within Eupsilobiinae.

### Zoogeography

New taxa described and collection localities increase the known distribution of African Eupsilobiinae from the Western Cape region to the Kruger National Park in the northeast and Natal Province in the southeast.

The range of *Microxenus* includes a western coastal area of Western Cape and Kruger National Park in northeastern South Africa. All three species appear to be allopatric. *Microxenus laticollis* is known only from the Cape of Good Hope; *M. muelleri* collected from a few costal localities of Western Cape between 31° 39′ N and 32°49′ S; *M. krugeri* collected at two localities, close to each other: 24° 13′ S, 31° 56′ E and 24° 59′ S, 31° 39′ E in Kruger National Park. *Natalinus* is known so far from a single locality in southern part of the Kwazulu-Natal Province (30° 34′ S, 29° 43′ E).

### Biology

*Microxenus laticollis* has been collected from or in the vicinity of ant nests (Pakaluk and Ślipiński 1990), and like several other species of Eupsilobiinae is probably a social insect inquiline. However, it is most probably mycophagous, feeding on a combination of spores and hyphae of microfungi (Skelley and Leschen 2002). The nature of the relationship between Eupsilobiinae and their social insect hosts remains unclear (Shockley et al. 2009a).

The specimens of *M. krugeri* and *M. muelleri* were collected from groundtraps with meat and banana bait. Additionally, *M. muelleri* was collected from groundtraps with feces bait. Attraction to meat and feces is unusual, and so far an unknown habit for endomychids.

*Natalinus klimaszewskii* was collected from sifted forest litter in a stinkwood forest *(Ocotea bullata).*
